# Indoor Environmental Quality (IEQ): A Comparison between TOPSIS- and PROMETHEE-Based Approaches for Indirect Eliciting of Category Weights

**DOI:** 10.3390/toxics11080701

**Published:** 2023-08-14

**Authors:** Francesco Lolli, Antonio Maria Coruzzolo, Elia Balugani

**Affiliations:** 1 Department of Sciences and Methods for Engineering, University of Modena and Reggio Emilia, Via Amendola 2, Padiglione Morselli, 42122 Reggio Emilia, Italy; francesco.lolli@unimore.it (F.L.); elia.balugani@unimore.it (E.B.); 2 En&Tech Interdipartimental Center, University of Modena and Reggio Emilia, Via Amendola 2, Padiglione Morselli, 42122 Reggio Emilia, Italy

**Keywords:** indoor environmental quality, IEQ, indirect eliciting, TOPSIS, PROMETHEE, AHP

## Abstract

Indoor Environmental Quality (IEQ) has received a great deal of attention in recent years due to the relationship between worker comfort and productivity. Many academics have studied IEQ from both a building design and an IEQ assessment perspective. This latter line of research has mostly used direct eliciting to obtain weights assigned to IEQ categories such as thermal comfort, visual comfort, acoustic comfort, and indoor air quality. We found only one application of indirect eliciting in the literature. Such indirect eliciting operates without the need for imprecise direct weighing and requires only comfort evaluations, which is in line with the Industry 5.0 paradigm of individual, dynamic, and integrated IEQ evaluation. In this paper, we use a case study to compare the only indirect eliciting model already applied to IEQ, based on TOPSIS, to an indirect eliciting method based on PROMETHEE and to a classical direct eliciting method (AHP). The results demonstrate the superiority of indirect eliciting in reconstructing individual preferences related to perceived global comfort.

## 1. Introduction

The importance of Indoor Environmental Quality (IEQ) has grown substantially in recent years, as it has been demonstrated that worker comfort impacts productivity [1]. Poor IEQ reduces self-reported work performance, measured cognitive performance, and well-being of office workers by lowering their attention and motivation while enhancing their tiredness [2]. From these findings, academics have designed architectures capable of maximizing occupant comfort [3] while ensuring buildings’ sustainability and efficiency [4], developed methods to assess IEQ and gauge differences in its evaluation, and developed models to capture the relationship between occupant comfort and IEQ categories [5]. The IEQ is obtained from categories that are evaluated individually, then aggregated into a final score. These categories usually include [6] Thermal Comfort (TC), Visual Comfort (VC), Acoustic Comfort (AC), and Indoor Air Quality (IAQ). However, other categories can be included as well, such as water quality, vibrations, and electromagnetic field (EM) [7]. This process usually involves multiple proxy variables for each category to be combined into the final IEQ evaluation. Many papers have studied the underlying relationship between these proxy variables and individual comfort, applying models such as linear regression [8], nonlinear regression [9], PCA [10], and Analytic Hierarchic Process (AHP) [11]. All these analyses took place in a Post-Occupancy Evaluation (POE) framework [12], in which practitioners collect environmental variables related to AC, VC, TC, and IAQ as well as the occupants’ personal preferences through standardized surveys [13]. For a full review of POE applications with a qualitative and quantitative assessment of the main factors impacting the IEQ, interested readers can refer to [14]. Most applications following a POE framework to assess the IEQ have applied direct eliciting, assuming that a decision-maker such as the operator or the building occupant can effectively express preferences between the categories involved. When AHP is applied, for example, an operator is asked to make pairwise comparisons between different categories based on Saaty’s scale [15,16]. Making such a comparisons among many categories can be cognitively demanding, an approach that is antithetical to the human-centric concept of Industry 5.0 [17], which strives to reduce operators’ cognitive effort while maximizing their comfort. To limit this cognitive effort, indirect eliciting has been introduced to the field of MCDM [18,19] following this new industrial revolution; it only requires the operator to evaluate a few experienced situations in terms of general comfort without expressing direct preferences over IEQ categories. To the best of our knowledge, only one previous contribution has applied indirect eliciting to the IEQ evaluation [20], in which case the authors proposed an approach based on the Technique for Order of Preference by Similarity to Ideal Solution (TOPSIS) [21]. The scope of this paper is to apply the same indirect eliciting approach to a case study and compare its performance in terms of reconstructed preferences with a different indirect eliciting approach based on the Preference Ranking Organization METHod for Enrichment of Evaluations (PROMETHEE). In particular, we applied the method proposed in [22], which has never been applied to an IEQ evaluation before, and compared both indirect eliciting methods to classic AHP direct eliciting [15,23]. The rest of this paper is structured as follows: Section 2 provides a literature review of IEQ evaluation with a focus on direct and indirect methods; Section 3 provides the mathematical foundation for the applied models; Section 4 details the case study; and Section 5 presents our conclusions and future research agenda.

## 2. Literature Review

IEQ, as per the American Society of Heating Refrigeration and Air Conditioning Engineers definition, is “a perceived indoor experience about the building indoor environments that includes aspects of design, analysis, and operation of energy efficient, healthy and comfortable buildings” [24]. IEQ has been studied extensively in many of its aspects, such as occupant acceptance [25,26,27], productivity [27], dynamic Life Cycle Assessment (LCA) [28], and green building performance [29]. The IEQ is evaluated by collecting different types of data to be aggregated differently depending on the specific method used. These data can be objective, in which case they refer to physical measurements (proxy IEQ variables); obtained in a controlled environment, in which case they relate to the main four IEQ categories (TC, VC, AC, IAQ); as-is; or through derived variables. Thermal comfort evaluation, for example, is usually carried out following the UNI-EN ISO 7730:2006 standard [30], which requires evaluation of the Predicted Mean Vote (PMV) and Predicted Percentage of Dissatisfied (PPD) following Fanger’s method [31]. To obtain these derived variables, environmental IEQ variables such as air temperature, relative humidity, mean radiant temperature, and relative air velocity must be collected. At the same time the method requires the collection of individual IEQ variables such as clothing insulation and metabolic activity. With these inputs, a derived variable can be calculated for each operator or occupant. For other categories different environmental and individual IEQ variables can then be collected to provide a unique IEQ evaluation. Subjective variables related to the occupants’ level of satisfaction and comfort can be collected; in this case, IEQ models look for correlations between the objective IEQ variables in each category and the occupants’ subjective comfort levels. IEQ models can be categorized based on the type of method exploited to find this correlation [32], allowing IEQ models to be classified based on subjective–objective and objective criteria. The first category refers to IEQ evaluations in which subjective measurement are collected from operators and occupants and in which relationships between objective measurements are investigated. Objective criteria models, on the other hand, do not collect any subjective measurements, and rely only on the collection of objective variables. In this type of IEQ model, the category evaluation is obtained by comparing measured values with a pre-defined set of criteria to obtain a class for each category, such as “healthy”, “non-healthy”, or “uncertain” [33]; then, a final IEQ evaluation with different weighting methods is obtained. Within this framework assessment, class limits, which are the thresholds that link a class to a category, are considered controversial, and vary widely among different studies [32]. For this reason, and because our research is more in line with the subjective–objective class of models, we focus our review on this stream.

In subjective–objective IEQ methods, the final score is calculated by weighting the different categories using, for example, multivariate regression [5] to link occupants comfort with controlled variables. A common approach of this class of models is the use of surveys to collect perceived comfort at both the category level and overall; then, the relationships between controlled variables and category comfort and between category comfort and overall comfort are investigated.

The two different relationships can be found using the same or different mathematical models, and can be defined as follows:

1. R_a_: the relationship between the perceived comfort in each category (AC, VC, TC, IAQ) and the controlled variables in each category.
2. R_b_: the relationship between the final IEQ evaluation and the comfort of each category in the weighting of IEQ categories (note that not all papers account for a weighting procedure).

For both R_a_ and R_b_, different types of regressions and MCDM can be applied. In this line of research, one of the first published papers [8] included a POE protocol with a real-time survey as well as a collection of environmental variables. The authors weighted different criteria equally (i.e., no weighting for R_b_) and investigated R_a_ using a linear regression, one of the most common methods for this scope. Using a similar approach, Mui and Chan [34] exploited a linear regression for R_a_, while R_b_ was investigated using multiple linear regression on TC, AC, IAQ, and VC. Following the trend of using regressions to interpret R_a_ and R_b_, in [35], as in [34], the authors applied a linear regression for R_a_ while exploiting a logistic regression for R_b_. In these papers the same type of regression was used for all the categories involved in R_a_; however, this is not a general rule. In other papers the authors have exploited different models for R_a_ in the different categories, suggesting that the relationship between controlled variables and comfort depends on the comfort category considered. In [5], for example, the authors exploited a linear regression for AC and IAQ while applying a nonlinear regression to TC and VC. At the same time, the authors applied multiple nonlinear regression for R_b_. In a similar way, in [9] the authors exploited a linear regression for VC and nonlinear regressions for AC, TC, and IAQ while a multiple nonlinear regression was used to fit R_b_. Another stream of research in this field does not use regression, instead applying MCDM for R_a_ and/or R_b_. In Chiang and Lai [33] the authors exploited AHP to weight both essential indicators in each IEQ category, R_a_, and to weight the IEQ categories themselves, R_b_. In a similar way, in [11] the authors applied AHP to R_b_ to derive the weights for IEQ categories. All the cited papers applying MCDM have exploited a direct eliciting approach. This is true for MCDM applied to Occupational Health and Safety Risk Assessment (OHSRA), where the use of MCDM is common practice [36], as demonstrated in [37]. To the best of our knowledge, only in [20] have the authors applied indirect eliciting for IEQ evaluation. They found individual weights for the IEQ categories using indirect eliciting based on TOPSIS, and demonstrated the superiority of indirect eliciting compared to AHP in reconstructing operator/occupant preferences. The lack of application of indirect eliciting to IEQ is unexpected, as different types of relations have been tested to interpret individual perceived comfort, while an alternative requiring less cognitive effort has been mostly ignored. Here, in line with the only work that has exploited indirect eliciting, we test the indirect eliciting method proposed in [20] against a modification of the indirect eliciting method applied in [38,39,40] and against classical AHP as a benchmark for the most common direct eliciting method.

## 3. Mathematical Models

In this section, we present the mathematical models applied in the case study; Section 3.1 presents the indirect eliciting approach based on TOPSIS, while Section 3.2 presents the indirect eliciting based on PROMETHEE. Each model focuses on K IEQ variables associated with T time steps, defined as $S_{kt}$.

### 3.1. TOPSIS

Classic TOPSIS [21,41] uses the following algorithm for cost criteria:

1. The risk variables $S_{kt}$ are normalized to obtain normalized risk variables for each time step $n_{kt}$:
  (1) $$n_{kt}=\frac{S_{kt}}{\sqrt{\sum_{t=1}^{T}S_{kt}^{2}}}\;\forall k=1,\ldots ,K\;\forall t=1,\ldots ,T$$
2. The unweighted ideal $a_{k}^{+}$ and anti-ideal solution $a_{k}^{-}$ are computed for each IEQ variable:
  (2) $$a_{k}^{+}=min\left(n_{k1},\ldots ,n_{kt}\right)\;\forall k=1,\ldots ,K$$
  (3) $$a_{k}^{-}=max\left(n_{k1},\ldots ,n_{kt}\right)\;\forall k=1,\ldots ,K$$
3. The normalized risk variables are weighted to obtain weighted normalized risk variables for each time step $c_{kt}$:
  (4) $$c_{kt}=w_{k}\cdot n_{kt}\;\forall k=1,\ldots ,K\;\forall t=1,\ldots ,T$$
  where the weights must add up to one:
  (5) $$\sum_{k=1}^{K}w_{k}=1$$
4. The Euclidean distances from the ideal $d_{t}^{+}$ and anti-ideal $d_{t}^{-}$ solutions are:
  (6) $$d_{t}^{+}=\sqrt{\sum_{k=1}^{K}{\left(c_{kt}-w_{k}\cdot a_{k}^{+}\right)}^{2}}$$
  (7) $$d_{t}^{-}=\sqrt{\sum_{k=1}^{K}{\left(c_{kt}-w_{k}\cdot a_{k}^{-}\right)}^{2}}$$
5. The final IEQ values $r_{t}$ for each time step are:
  (8) $$r_{t}=\frac{d_{t}^{-}}{d_{t}^{+}+d_{t}^{-}}$$

In our hypothesis, the IEQ categories weights $w_{k}$ are unknown and the eliciting procedure optimizes them by solving the following maximization problem [20] where, for each time step, a subjective IEQ evaluation has been collected $\left[\begin{array}{c} S_{1t}\\ \ldots \\ S_{Kt} \end{array}\right]≻\left[\begin{array}{c} S_{1t^{\prime }}\\ \ldots \\ S_{Kt^{\prime }} \end{array}\right]$:

(9) $$max\sum_{\left[\begin{array}{c} S_{1t}\\ \ldots \\ S_{Kt} \end{array}\right]≻\left[\begin{array}{c} S_{1t^{\prime }}\\ \ldots \\ S_{Kt^{\prime }} \end{array}\right]}ln\left(\frac{1}{1+e^{-\left(r_{t}-r_{t^{\prime }}\right)}}\right)$$

s.t.

(10) $$0\leq w_{k}\;\forall k=1,\ldots ,K$$

Such an optimization problem does not scale the weights, which must be corrected in order for them to add up to one:

(11) $$w_{k}≔\frac{w_{k}}{\sum_{k=1}^{K}w_{k}}\;\forall k=1,\ldots ,K$$

### 3.2. PROMETHEE

Classic PROMETHEE [39] adheres to the following algorithm for benefit criteria:

1. The differences $d_{ktt^{\prime }}$ between risk variables in different time steps ($S_{kt}$ and $S_{kt^{\prime }}$ for time steps t and $t^{\prime }$) are computed for each IEQ category K:
  (12) $$d_{ktt^{\prime }}=S_{kt}-S_{kt^{\prime }}\;\forall t=1,\ldots ,T\;\forall t^{\prime }=1,\ldots ,T$$
2. Each difference is transformed through a different preference function for each IEQ category to obtain $p_{ktt^{\prime }}$:
  (13) $$p_{ktt^{\prime }}=f_{k}\left(d_{ktt^{\prime }}\right)$$
  With $p_{ktt^{'}}\in \left[0,1\right]$.
  In this paper, the preference function is linear:
  (14) $$p_{ktt^{\prime }}=\left\{\begin{array}{cc} 1 & if\;d_{ktt^{\prime }}\geq {max}_{t,t^{\prime }}\left(d_{ktt^{\prime }}\right)\\ \frac{{max}_{t,t^{\prime }}\left(d_{ktt^{\prime }}\right)-d_{ktt^{\prime }}}{{max}_{t,t^{\prime }}\left(d_{ktt^{\prime }}\right)} & if\;0\leq d_{ktt^{\prime }}\leq {max}_{t,t^{\prime }}\left(d_{ktt^{\prime }}\right)\\ 0 & if\;d_{ktt^{\prime }}\leq 0 \end{array}\right.$$
3. The transformed differences are weighted over the IEQ categories to obtain a single $s_{tt^{\prime }}$ for each pair of time steps t and $t^{\prime }$:
  (15) $$s_{tt^{\prime }}=\sum_{k=1}^{K}w_{k}\cdot p_{ktt^{\prime }}$$
4. Leaving $\phi_{t}^{-}$ and entering $\phi_{t}^{+}$, flows are then computed for each time step:
  (16) $$\phi_{t}^{+}=\sum_{t^{\prime }=1}^{T}s_{tt^{\prime }}$$
  (17) $$\phi_{t}^{-}=\sum_{t=1}^{T}s_{tt^{\prime }}$$
5. The net flows $\phi_{t}$ are obtained as follows:
  (18) $$\phi_{t}=\phi_{t}^{+}-\phi_{t}^{-}$$

In our hypothesis, the weights are unknown and the eliciting procedure optimizes them by solving the following maximization problem, where a subjective IEQ evaluation has been collected for each time step $\left[\begin{array}{c} S_{1t}\\ \ldots \\ S_{Kt} \end{array}\right]≻\left[\begin{array}{c} S_{1t^{\prime }}\\ \ldots \\ S_{Kt^{\prime }} \end{array}\right]$:

(19) $$max\sum_{\left[\begin{array}{c} S_{1t}\\ \ldots \\ S_{Kt} \end{array}\right]≻\left[\begin{array}{c} S_{1t^{\prime }}\\ \ldots \\ S_{Kt^{\prime }} \end{array}\right]}ln\left(\frac{1}{1+e^{-\left(\phi_{t}-\phi_{t^{\prime }}\right)}}\right)$$

s.t.

(20) $$0\leq w_{k}\;\forall k=1,\ldots ,K$$

This methodology is similar to the one proposed in [38], and is computationally faster [22].

The two proposed models are general, and can be applied to other contexts to obtain indirectly elicited optimized weights able to reconstruct operator or occupant comfort based on the collected variables and on their global comfort perception.

## 4. Case Study

### 4.1. Data Collection

The case study took place in Reggio Emilia, Italy, in March 2023 and considered an administration office located in a research center with four operators. The office area is 5.68 m × 11.88 m and the operators work at the same shared central desk in an open environment. To obtain an IEQ evaluation, a set of four IEQ categories (AC, VC, TC, and IAQ) was defined and one or more proxy variables were collected for each category through low-cost sensors. Because low-cost sensors were exploited, only certain proxy variables for each IEQ category were collected. In particular, we collected the following variables for each category:

- TC: we collected the air temperature and relative humidity with a sensor placed 1.1 m from the ground and 1.5 m from the shared desk. In order to calculate PMV, which is the classic measure of comfort for TC as prescribed in the UNI-EN ISO standard 7730:2006 [30], the mean radiant temperature, relative air velocity, clothing insulation, and metabolic activity should all be collected. Regarding the mean radiant temperature, a default value of 25° was used, as the building is new and the walls are well insulated. For air velocity, a default value of 0.1 m/s was used, as the addendum to ASHRAE 55 suggests the use of the PMV model with air speeds below 0.20 m/s [40]. For the metabolic rate, a value of 1.2 met was used for all operators, with a clothing insulation level of 1.2 clo for everyone except Operator 3, whose clothing was lighter and equal to 1 clo.
- VC: we collected the desk surface illuminance.
- AC: we collected the A-weighted daily noise exposure with a phonometer placed 1.2 m from the ground.
- IAQ: we collected the pm2.5 concentration with a sensor 1.1 m from the ground and 1.5 m from the shared desk.

The IEQ variables collected for each IEQ category and the sensor characteristics (unit of measure, resolution, and measurement range) are summarized in Table 1.

### 4.2. Data Analysis

**Figure 1:**
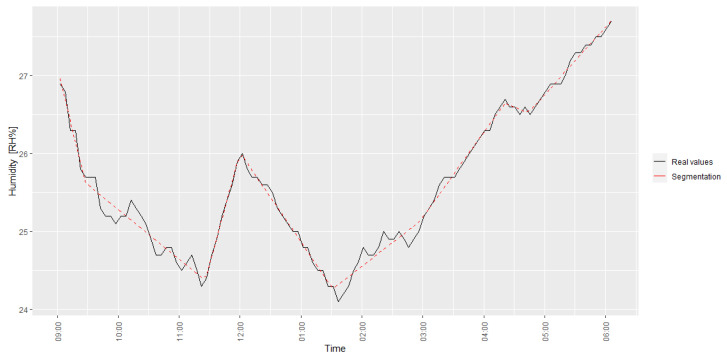
Humidity IEQ variable segmentation.

The environmental working conditions of the four operators were monitored for 8 h, with a one-hour lunch break. The frequency of this collection process was high (e.g., one second) and continued for the whole duration of the analysis. A time step of one hour was selected and piece-wise linear segmentation [41,42] was implemented to obtain segments from these high-frequency data; this process removes most of the data variability. The mean value of each segmented variable was associated with its corresponding time step and the IEQ variables referring to the same IEQ category were transformed into $S_{kt}$ IEQ-derived variables, where k is the IEQ category and t is the time step. The humidity (one of the TC variables), for example, was collected once every second; this high-frequency IEQ variable was then segmented thorough piece-wise linear regression, and the mean value of this segmented function was averaged for each hour t. As an example, Figure 1 shows this segmentation process applied to humidity for the day under evaluation. This process was repeated for the temperature and the two IEQ variables were transformed for a given an operator’s features into a single derived IEQ variable (PMV) associated with the TC IEQ category. This process was replicated for all the other IEQ variables to obtain an $S_{kt}$-derived variable for each IEQ category k and time step t. If an IEQ category was associated with a single IEQ variable, that variable was used after the segmentation and averaging process instead of a derived variable.

Each IEQ (derived) variable was associated with an acceptability range obtained from different Italian standards; however, this range can be adapted to different regulations depending on the county where the evaluation takes place:

- The pm_2.5_ concentration should fall below 40 $\frac{\mu g}{m^{3}}$ [43].
- The PMV is dimensionless, and should fall between −2 and 2, with zero representing comfort [30].
- The desk surface illuminance should be at least 300 lux for filling and copying activities [44].
- The A-weighted daily noise exposure level should fall below 87 dB [45].

Considering these ranges, the $S_{kt}$ values cannot be analyzed as-is, and must be transformed as follows:

- The pm_2.5_ concentration is used as-is.
- The PMV is substituted by its distance from 0 in absolute value.
- The desk surface illuminance is reduced by 300 lx, and this distance is then used as an absolute value.
- The A-weighted hourly noise exposure level is used as-is.

Following these transformations, all the IEQ (derived) variables must be minimized.

For each time step t, each operator provides a subjective IEQ evaluation on a scale from 1 to 5, where 5 represents ideal conditions. These evaluations are used to order the different time steps comfort values:

(21) $$\;\left[\begin{array}{c} S_{1t}\\ \ldots \\ S_{Kt} \end{array}\right]≻\left[\begin{array}{c} S_{1t^{\prime }}\\ \ldots \\ S_{Kt^{\prime }} \end{array}\right]$$

where, in this case, the operator ranked the comfort in time step t higher than that in time step $t^{\prime }$.

These relations are used with the mathematical methods introduced in the previous section to obtain a weight $w_{i}$ for each IEQ (derived) variable. These weights are necessary to evaluate new IEQ (derived) variables values without the need for the operator’s intervention.

The collected variables led to the hourly IEQ variables in Table 2; shared variables are only reported once, while the PMV is computed individually for each operator.

**Figure 2:**
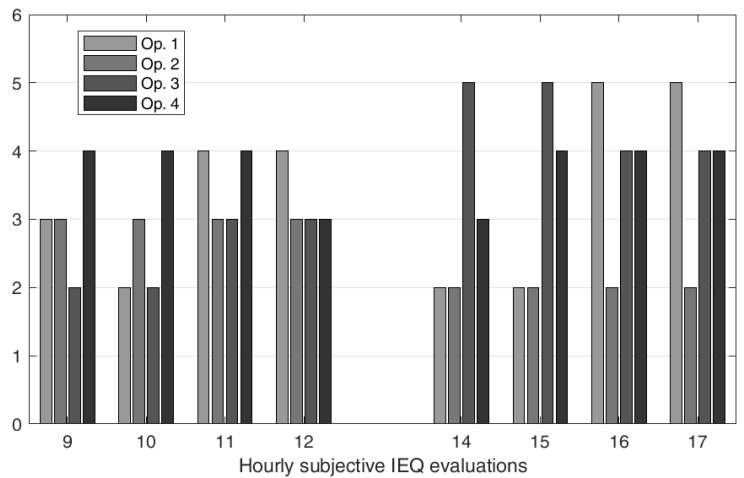
Hourly subjective IEQ evaluations.

Every hour, each operator provided a subjective global comfort evaluation, shown in Figure 2, ranking their global comfort from 1 to 5, where 5 represents ideal conditions. These subjective evaluations were required for both the indirect eliciting and to analyze the ability to reconstruct the operators’ preferences.

In addition, at the end of the 8 h each operator filled in an AHP preference matrix comparing the IEQ categories (Table 3, Table 4, Table 5 and Table 6) in order to obtain directly elicited subjective categories weights. This direct eliciting is only needed as a benchmark with the indirect eliciting approach, which does not require it. In total, each operator provides six comparisons, for a total of 24 subjective comparisons among IEQ categories for the four operators.

From these data, following the mathematical models’ algorithms reported in Section 3.2, we obtained the following:

- Four sets of weights from the AHP, one for each operator from their AHP preference matrix.
- Four optimized sets of weights for TOPSIS, one for each operator from the hourly IEQ (derived) variables and the subjective evaluations on global comfort.
- Four optimized sets of weights for PROMETHEE, one for each operator from the hourly IEQ (derived) variables and the subjective evaluations on global comfort.
- To solve the optimization problems, the subjective IEQ evaluations were converted into nonredundant relations. For example, Operator 1 evaluated the hour 9 IEQ as 3, hour 10 IEQ as 2, and hour 11 IEQ as 4; thus, all possible relations are:

(22) $$\left[\begin{array}{c} S_{11}\\ S_{21}\\ S_{31}\\ S_{41} \end{array}\right]≻\left[\begin{array}{c} S_{12}\\ S_{22}\\ S_{32}\\ S_{42} \end{array}\right]$$

(23) $$\left[\begin{array}{c} S_{13}\\ S_{23}\\ S_{33}\\ S_{43} \end{array}\right]≻\left[\begin{array}{c} S_{12}\\ S_{22}\\ S_{32}\\ S_{42} \end{array}\right]$$

(24) $$\left[\begin{array}{c} S_{13}\\ S_{23}\\ S_{33}\\ S_{43} \end{array}\right]≻\left[\begin{array}{c} S_{11}\\ S_{21}\\ S_{31}\\ S_{41} \end{array}\right]$$

While $\left[\begin{array}{c} S_{13}\\ S_{23}\\ S_{33}\\ S_{43} \end{array}\right]≻\left[\begin{array}{c} S_{12}\\ S_{22}\\ S_{32}\\ S_{42} \end{array}\right]$ is redundant, and as such was not used in the optimization problems.

From these weights, we then computed the following:

- An IEQ evaluation for each operator and hour, using the AHP weights and the hourly IEQ (derived) variables in TOPSIS.
- An IEQ evaluation for each operator and hour, using the AHP weights and the hourly IEQ (derived) variables in PROMETHEE.
- An optimized IEQ evaluation for each operator and hour, using the optimized TOPSIS weights and the hourly IEQ (derived) variables in TOPSIS.
- An optimized IEQ evaluation for each operator and hour, using the optimized PROMETHEE weights and the hourly IEQ (derived) variables in PROMETHEE.

For each of these four methods it is possible to check how many of the nonredundant relations that we aimed to verify were upheld. Here, the ratio of nonverified relations is called the error rate, and is proposed in Table 7 for each operator and tested method.

**Figure 3:**
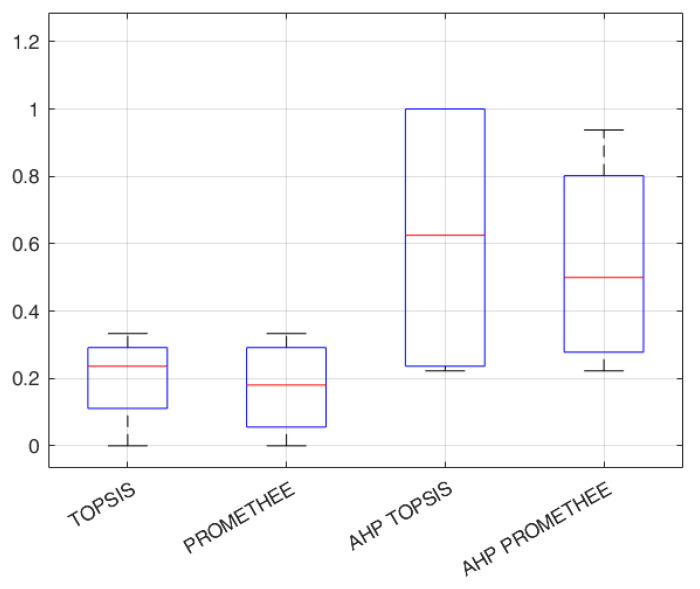
Error rates for different methods.

Even if the data are too limited for a statistical test, the directly elicited AHP weights in both TOPSIS and PROMETHEE provide substantially higher error rates than the indirectly elicited ones obtained through the optimized methods. A boxplot is depicted in Figure 3, where it can be seen that the variability in the error rates with the optimized methods is significantly less pronounced.

**Figure 4:**
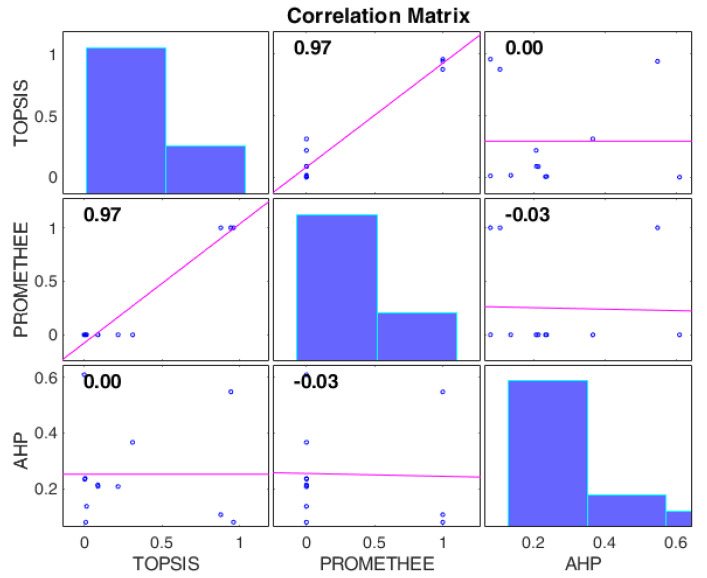
Correlations between weights of different methods.

Figure 4 shows a correlation matrix highlighting the correlation between methods and weights. The figure shows a strong positive correlation between optimized TOPSIS and PROMETHEE weights, while the AHP ones are uncorrelated. To obtain Figure 4, only the operators’ PMV, desk surface illuminance, and pm_2.5_ weights were used, while the A-weighted hourly noise exposure weight was discarded, as it was correlated with them by design (each set of weights adds up to 1).

## 5. Conclusions

In this paper, we applied two different indirect eliciting methods, one based on TOPSIS, presented in [20], and the other on PROMETHEE, never before applied in this field [22,38] to obtain individual weights for four IEQ categories. With these weights obtained, a dynamic and individual IEQ evaluation was determined for each operator and hour. This solution provides high flexibility, and thanks to the segmentation phase it can be used with multiple non-aligned sensors. Another important aspect of the proposed solution is the individuality of the IEQ criteria weights, which, in line with the human-centric paradigm of Industry 5.0 [46], takes into account personal preferences. These two indirect eliciting methods can be applied to different environments, industrial or not, following the proposed framework in which control variables are collected from different non-aligned sensors and personal preferences around overall comfort. Here, these methods were applied in a case study involving four different operators in an administrative office and tested against a classical direct eliciting method, namely, AHP. The case study considered all the relevant IEQ categories as per the literature: AC, TC, VC, and IAQ. The case study results show the superiority of the two indirect eliciting methods in reconstructing operator preference; thus, our findings can be summarized as follows:

- AHP weights are unreliable, and can result in very high error rates when reconstructing operators’ preferences (error rates of up to 100% for AHP-TOPSIS for two of the operators).
- Indirect elicited TOPSIS and PROMETHEE optimized weights provide similar high-quality results, with low error rates compared to AHP.
- The similarity between optimized TOPSIS and PROMETHEE results can be explained by the high correlation between their weights.

Future research in this direction could include testing of the proposed models on a larger scale involving more operators and more collection days as well as different environments and seasons, and the inclusion of other direct eliciting methods as a benchmark in addition to AHP. 

## Figures and Tables

**Table 1:** Collected IEQ variables and sensor characteristics.

IEQ Category	IEQ Variable	Unit of Measure	Resolution	Measurement Range
IAQ	pm_2.5_ concentration	$\frac{\mu g}{m^{3}}$	$1\;\frac{\mu g}{m^{3}}$	[0, 1000]
VC	Desk surface illuminance	lx	0.1 lx	[0, 120,000]
TC	Air temperature	°C	0.1 °C	[−40, 85]
	Relative Humidity	RH	0.1 RH	[0, 100]
AC	A-weighted equivalent continuous sound pressure level	dB	0.1 dB	[22, 136]

**Table 2:** Hourly IEQ (derived) variables.

Time	PMV Operator 1	PMV Operator 2	PMV Operator 3	PMV Operator 4	Desk Surface Illuminance	pm2.5	A-Weighted Equivalent Continuous Sound Pressure Level
9	0.191	0.191	−0.050	0.191	592.7	9.3	46.4
10	0.373	0.374	0.162	0.373	585.2	8.4	46.6
11	0.452	0.453	0.250	0.452	577.8	7.6	46.4
12	0.508	0.508	0.309	0.508	570.3	6.7	45.1
14	0.656	0.657	0.471	0.656	481.3	5.4	44.9
15	0.654	0.654	0.468	0.654	480.7	5.2	47.8
16	0.601	0.601	0.409	0.601	480.1	4.9	41.5
17	0.554	0.554	0.358	0.554	479.4	4.6	40.7

**Table 3:** Operator 1 AHP preference matrix.

	pm_2.5_	PMV	Desk Surface Illuminance	A-Weighted Equivalent Continuous Sound Pressure Level
**pm**_**2.5**_	1	3	$\frac{1}{3}$	$\frac{1}{3}$
**PMV**	$\frac{1}{3}$	1	1	2
**Desk surface illuminance**	3	1	1	3
**A-weighted equivalent continuous sound pressure level**	3	$\frac{1}{2}$	$\frac{1}{3}$	1

**Table 4:** Operator 2 AHP preference matrix.

	pm_2.5_	PMV	Desk Surface Illuminance	A-Weighted Equivalent Continuous Sound Pressure Level
**pm**_**2.5**_	1	4	4	$\frac{1}{5}$
**PMV**	$\frac{1}{4}$	1	1	$\frac{1}{5}$
**Desk surface illuminance**	$\frac{1}{4}$	1	1	$\frac{1}{5}$
**A-weighted equivalent continuous sound pressure level**	5	5	5	1

**Table 5:** Operator 3 AHP preference matrix.

	pm_2.5_	PMV	Desk Surface Illuminance	A-Weighted Equivalent Continuous Sound Pressure Level
**pm_2.5_**	1	$\frac{1}{5}$	3	3
**PMV**	5	1	5	5
**Desk surface illuminance**	$\frac{1}{3}$	$\frac{1}{5}$	1	2
**A-weighted equivalent continuous sound pressure level**	$\frac{1}{3}$	$\frac{1}{5}$	$\frac{1}{2}$	1

**Table 6:** Operator 4 AHP preference matrix.

	pm_2.5_	PMV	Desk Surface Illuminance	A-Weighted Equivalent Continuous Sound Pressure Level
**pm_2.5_**	1	$\frac{1}{4}$	3	3
**PMV**	4	1	4	4
**Desk surface illuminance**	$\frac{1}{3}$	$\frac{1}{4}$	1	3
**A-weighted equivalent continuous sound pressure level**	$\frac{1}{3}$	$\frac{1}{4}$	$\frac{1}{3}$	1

**Table 7:** Error rates for different methods.

	AHP TOPSIS	AHP PROMETHEE	TOPSIS	PROMETHEE
**Operator 1**	0.22	0.22	0.22	0.11
**Operator 2**	1	0.94	0	0
**Operator 3**	1	0.67	0.33	0.33
**Operator 4**	0.25	0.33	0.25	0.25

## Data Availability

Data available on request.
